# Hepatitis C infection, related services, and barriers to HCV treatment among drug users in methadone maintenance treatment (MMT) clinics in Shanghai, China

**DOI:** 10.1186/s12954-017-0197-3

**Published:** 2017-11-02

**Authors:** Zhi-Bin Li, Lei Zhang, Jun Wang, Le-Ping Huang, Zhi-Rong Zhou, Yi-Ning Cao, Min Zhao, Jiang Du

**Affiliations:** 1 Shanghai Mental Health Center, Shanghai Jiao Tong University School of Medicine, Shanghai, China; 2 Yangpu Mental Health Center, Shanghai, China; 3 Hongkou Mental Health Center, Shanghai, China; 4 Xuhui Mental Health Center, Shanghai, China; 5 Minhang Mental Health Center, Shanghai, China; 6 Jiading Mental Health Center, Shanghai, China; 7 Shanghai Key Laboratory of Psychotic Disorders, Shanghai, China; 8 Brain Science and Technology Research Center, Shanghai Jiao Tong University, Shanghai, China

**Keywords:** Hepatitis C, Methadone maintenance treatment, Heroin dependent, China

## Abstract

**Background:**

The purpose of this study was to document the prevalence of hepatitis C among MMT patients, hepatitis C virus (HCV) knowledge of patients and MMT staff members, and the barriers preventing them from receiving or delivering HCV-related services in MMT clinics of China.

**Methods:**

Data were collected from 240 MMT patients and 58 staff members in Shanghai MMT clinics. Structured questionnaires (HCV Knowledge Scale and Alcohol Use Disorders Identification Test) and several self-developed questionnaires were used to assess (1) patient and staff HCV knowledge, (2) attitudes toward HCV-related services in MMT clinics, and (3) what type of HCV-related services the staff members have provided in their routine work. The HCV test results were based on the patients’ medical records.

**Results:**

The HCV seropositive rate was high (70%), and both patients and staff had limited HCV knowledge. The mean score of patient HCV knowledge was 6.8 out of 20 (SD = 3.7), whereas the mean score of staff HCV knowledge was 10.9 out of 20 (SD = 3.1). For HCV-positive patients, only 13.7% had accessed HCV medical treatment. Barriers included the cost of medical treatment, lack of HCV knowledge, lack of professional training for patients to receive HCV-related services from individuals or MMT clinics, and lack of an adequate policy-making system.

**Conclusions:**

HCV infection remains an important problem among MMT patients in China. Barriers to HCV-related services are attributable to individual, clinical, and policy-related factors. This study may provide evidence-based information for future work to optimize the resources of MMT clinics.

**Trial registration:**

ClinicalTrials.gov NCT01647191. Registered 17 April 2012.

## Background

Hepatitis C virus (HCV) infection is a serious public health problem worldwide. The World Health Organization (WHO) has estimated that 185 million people are chronically infected with HCV globally, and at least 3.7% of East Asian people are HCV positive [1]. With an estimated population of 1.3 billion, the number of HCV-infected people in China could exceed 48 million. HCV infection has become one of the most important causes of chronic hepatitis, cirrhosis, and hepatocellular carcinoma in China [2].

High-risk factors for HCV infection include injection drug use (IDU) and transfusion of blood products. Since the establishment of a security system for blood donors in China, IDU has been the predominant mode of HCV transmission. By the end of 2014, approximately 2.96 million drug users were documented in China [3]. HCV infection has become a major health burden in China with rapid transmission, a long duration of clinical progression, and limited access of infected addicts to routine treatment.

Despite the high rate of HCV infection among IDUs, the majority of drug users opt out of HCV services, and few engage in antiviral treatment for HCV compared to non-drug users [4–6]. To address the escalating HCV problem in IDU, the Chinese government conducts routine HCV testing in methadone maintenance treatment (MMT) clinics and provides HCV-related training to MMT staff by the National MMT Training Center. However, this is not a sufficient training. Based on our previous study, drug users in MMT clinics had limited HCV knowledge, and most of them did not know their HCV status [7, 8].

Drug abuse treatment programs have been defined as a good platform to deliver HCV/HIV-related interventions or medical services. As a strategy to solve the problems of opiate abuse and HIV, the Chinese government established MMT clinics throughout the country beginning in 2004. By the end of 2011, there were 738 MMT clinics in the entire country, and 300,000 heroin users benefited from this service [9]. MMT clinics have been effective in preventing the spread of HIV/AIDS [10, 11]. Given the same transmission route and the Chinese situation, incorporating HCV prevention/intervention strategies into the MMT setting could be performed more fully and effectively to prevent HCV-related consequences and increase medical uptake.

However, no study has yet reported on HCV-related services and barriers for patients to receive HCV treatment among drug users in China. Therefore, the first step is to obtain information about (1) patient and staff knowledge, (2) attitudes toward HCV-related services in MMT clinics, and (3) what type of HCV-related service the staff have provided in their routine work. Therefore, we conducted this study (1) to document the prevalence of hepatitis C among MMT patients, (2) to explore HCV knowledge and attitudes toward HCV services among patients and MMT staff, and (3) to investigate the HCV-related services provided by treatment staff and identify the barriers that prevent patients and treatment staff from using or providing related services in the clinics.

## Methods

### Study participants

#### MMT patients

There were 14 MMT clinics in Shanghai located in urban areas. Since the final aim of the proposal was to evaluate the effectiveness of HCV/HIV comprehensive interventions among drug users in MMT clinics, we defined any MMT clinic with more than 100 patients as meeting the inclusion criteria. There were nine MMT clinics that met the criteria, and four of them were randomly selected. At each selected clinic, the study was explained to potential participants during the recruitment process. For those who decided to participate in this study, detailed informed consent procedures outlined the nature of participation, risks, and benefits.

The patient eligibility criteria included opiate dependence (according to the criteria established by the Diagnostic and Statistical Manual IV, DSM-IV), at least 20 years of age or older, and being local residents. Sixty patients were randomly selected at each clinic for a total of 240 patients. All participants provided informed consent and were paid for their time.

The study participants were asked to respond to questions involving their socio-demographics, risk behaviors, and attitudes towards HCV-related services. They also provided consent to share clinical records and medical test results including drug use and HAV, HBV, and HCV. The research staff provided support for participants who may have had challenges with comprehending study procedures or questionnaires.

#### MMT staff

Drug abuse treatment staff who currently work at Shanghai MMT clinics were invited to participate in this survey via flyers posted in their offices and mailed to their institutes. To be eligible, staff had to have been working in the MMT program for at least 1 year. There were no other screening criteria for service providers. A total of 58 MMT staff were invited from 14 MMT clinics in Shanghai.

The research protocol was approved by the Ethics Committee of the Shanghai Mental Health Center, and each subject signed the informed consent form approved by IRB at the Shanghai Mental Health Center.

### Instruments and measures

Demographics for both patients and MMT staff and drug use history for patients were collected via a self-developed questionnaire.

#### Risk behaviors survey

An abbreviated version of the risk behaviors assessment developed by the National Institute on Drug Abuse (NIDA) was used; it is used to measure the risk behaviors related to HIV and HCV. Risk behaviors in the areas of drug use and sex in the previous 30 days were measured. Reliability and validity assessments of the risk behaviors survey (RBS) support its adequacy as a research tool for populations of drug users [12–14].

#### HCV knowledge questionnaire

HCV knowledge questionnaire includes 20 items concerning HCV transmission risk and risk behaviors, HCV diagnosis and disease progression, current HCV treatment options, and treatment outcomes. One point is given for each correct answer with a possible score ranging from 0 to 20. The instrument was translated into Chinese by one psychiatrist and back-translated by another psychiatrist, and it has been used in other studies with the same background [15–17].

#### The Alcohol Use Disorder Identification Test

The Alcohol Use Disorder Identification Test (AUDIT) was developed by the WHO to identify hazardous and harmful alcohol use and dependence, and it was specifically designed for international use [18]. This questionnaire consists of ten items to measure the quantity and frequency of alcohol use, possible dependence symptoms, and recent and lifetime problems associated with alcohol use. AUDIT was translated and validated in Chinese [19]. Alcohol consumption is an important factor in HCV infection prevention and treatment. Alcohol use may increase the risk of engaging in unprotected sex behaviors but can also exacerbate steatosis.

#### HCV-related services survey (for patients)

Perceptions of patients regarding HCV services were evaluated. The patients were asked to respond to a series of questions concerning HCV-related education, HCV/HIV testing, and medical support services in their current treatment programs. They were asked to indicate the extent on a scale from 1 (do not agree at all) to 10 (completely agree) concerning the reason for using these services. Some of questions required a “Yes” or “No” response.

#### HCV-related services survey (for MMT staff)

A self-developed questionnaire was used to collect information of HCV-related services provided by their treatment programs. It included HCV-related training, HCV testing, and HCV medical treatment support. All questions had “Yes” or “No” as responses.

#### Beck Depression Inventory

Depressive symptoms were screened with the Chinese version of the 21-item Beck Depression Inventory. Each item is scored from 0 to 3 with a maximum score of 63 [20]. In this study, Beck Depression Inventory (BDI) was used to investigate the depression situation among MMT patients.

HCV tests are part of the routine procedure in MMT clinics in China. HCV serostatus was tested by enzyme-linked immunosorbent assay (ELISA) in there. We obtained HIV, HAV, HBV, and HCV test results from the MMT clinic records with the permission of the participants and the clinic.

### Statistical analyses

Statistical Product and Service Solutions 20.0 (SPSS 20.0) was used to conduct the statistical analyses. Continuous data are presented as the mean and SD. Between-group differences for continuous variables were evaluated using Student’s *t* test. Between-group comparisons for categorical variables were performed using the chi-square test or Fisher’s exact test when necessary.

## Results

### Characteristics of the participants and hepatitis prevalence

The demographics of the participants and prevalence of hepatitis are summarized in Table 1. Overall, 79.6% of the participants were male. The mean age was 42.0 (8.6) years, and nearly half of the participants were married or living with partners. Over half (52.1%) had a middle school degree or less. There were no significant differences between HCV-positive and HCV-negative patients in those demographic characteristics.

**Table 1:** Demographic and hepatitis prevalence by HCV status

	Total (*n* = 240)	HCV positive (*n* = 168)	HCV negative (*n* = 72)
Gender, *n* (%)			
Males	191 (79.6)	132 (78.6)	59 (81.9)
Females	49 (20.4)	36 (21.4)	13 (18.1)
Age, mean (SD)	42.0 (8.6)	41.2 (8.5)	43.8 (8.7)
Age, *n* (%)			
21–35	70 (29.3)	56 (33.3)	14 (19.7)
36–50	127 (53.1)	88 (52.4)	39 (54.9)
51–61	42 (17.6)	24 (14.3)	18 (25.3)
Marital status, *n* (%)			
Never married/single	72(30.0)	52 (30.9)	20 (27.8)
Married/living together	115(47.9)	85 (50.6)	30 (41.7)
Divorced/separated	53(22.1)	31 (18.5)	22 (30.6)
Education, *n* (%)			
Middle school or less	125 (52.1)	88 (52.4)	37 (51.4)
High school or higher	115 (47.9)	80 (47.6)	35 (48.6)
Self-reported HCV test results, *n* (%)			
No	84 (35.0)	39 (23.2)	45 (62.5)
Yes	116 (48.3)	104 (61.9)	12 (16.7)
Unknown	40 (16.7)	25 (14.9)	15 (20.8)
HAV (IgG) positive, *n* (%)	104 (43.7)	76 (45.5)	28 (39.4)
HBV positive, *n* (%)*	20 (8.3)	10 (5.9)	10 (13.9)
HIV	0	0	0

*p < 0.05

A total of 70% of patients tested were positive for HCV. Among those tested positive, 38.1% reported their HCV status as negative or unknown. For HCV-negative patients, 16.7% reported that their test results were positive. Additionally, 8.3% of the participants were HBV positive. Participants who were HCV negative were significantly more likely to be infected with HBV compared to those who were HCV positive (*χ* ^2^ = 4.156, *p* = 0.041). No participants were infected with HIV.

### HCV knowledge and risk factors

The 240 participants answered an average of 6.8 out of 20 items correctly in the HCV knowledge assessment, which demonstrated a poor HCV knowledge level among this population (Table 2). There were no significant differences in HCV knowledge between the HCV-positive and HCV-negative groups. Regarding the drug use, injection, and sexual risk behavior history, the average period of drug use was 10.9 (6.7) years. HCV-positive patients had used drugs for a mean of 11.4 (7.2) years, which was significantly longer than the 10.0 (5.3) years of drug use reported by HCV-negative patients (*Z* = 1.674, *p* = 0.047). Few (2.6%) patients reported injection drug use in the past 30 days, and no one reported sharing injection needles. Almost 10% (9.6%) of the patients reported multiple sexual partners in past 30 days. More HCV-negative patients reported rarely or never using condoms with their casual or commercial sex partners compared to HCV-positive patients.

**Table 2:** HCV knowledge and risk behaviors by HCV status

	Total (*n* = 240)	HCV positive (*n* = 168)	HCV negative (*n* = 72)
HCV knowledge, mean (SD)	6.8 (3.7)	7.0 (3.5)	6.4 (3.8)
Time of drug use (year), mean (SD)*	10.9 (6.7)	11.4 (7.2)	10.0 (5.3)
Injection in past 30 days, *n* (%)	6 (2.6)	6 (3.8)	0 (0.0)
Shared needles in past 30 days, *n* (%)	0	0	0
More than one sexual partner in past 30 days, *n* (%)	10 (9.6)	7 (9.5)	3 (9.6)
Not using condoms when having sex, *n* (%)			
With primary sex partner	68 (66.0)	47 (63.5)	21 (72.4)
With casual sex partner	14 (14.0)	9 (12.3)	5 (18.5)
With commercial sex partner	12 (12.0)	6 (8.3)	6 (21.4)
Harmful drinking, *n* (%)	20 (8.3)	15 (8.9)	5 (6.9)
Current health is poor by self-report, *n* (%)	181 (75.7)	131 (78.5)	50 (69.5)
Depression symptoms in past 6 months, *n* (%)	109 (45.4)	79 (47.0)	30 (41.7)
Suicide ideation in past 6 months, *n* (%)	39 (16.3)	30 (17.9)	9 (12.5)
BDI > 15, *n* (%)	107 (44.9)	72 (43.4)	35 (48.6)

Note 1: Individuals who did not answer the sexual risk questions were dropped from the analysis. Thus, among the 240 participants, 104 were not included in the more than one sexual partner in past 30 days question, 103 were not included in the condom use with primary sex partner question, 100 were not included in the condom use with casual sex partner question, and 100 were not included in the condom use with commercial sex partner question

Note 2: Harmful drinking is defined by an AUDIT score of 7 or more

*p < 0.05

In this study, 15% of patients reported drinking alcohol during the last year, and 8.3% met the criteria of harmful drinking. HCV-positive patients displayed more harmful drinking behavior than HCV-negative patients, but the difference was not significant.

Overall, three quarters of the patients had a self-reported poor health situation, and 45.4% had been depressed in the past 6 months. In total, 44.9% of the participants experienced mild depression or more severe depression symptoms in the last week according to the BDI and 16.3% expressed suicide notions. HCV-positive participants were more likely to have suicide notions compared to HCV-negative participants.

### Patients’ perceptions of HCV-related services

When asked the rate of their needs for HCV education and medical support, the patients assigned scores of 6.0 (3.1) and 7.1 (2.9), respectively. For HCV testing services, 82.3% of the participants admitted that an HCV test is necessary to receive the treatment. Table 3 describes the participants’ responses about barriers that prevent them from using HCV services. Above all, the patients reported that they really appreciated the HCV services provided in current treatment programs. However, only 13.7% of HCV-positive patients accessed HCV medical treatment based on their self-report.

**Table 3:** Patients’ perception of HCV-related services in their treatment programs

	Total (*n* = 240)	HCV positive (*n* = 168)	HCV negative (*n* = 72)
Need HCV-related training when receiving treatment, mean (SD)	6.0 (3.1)	6.1 (3.2)	5.9 (3.2)
Need HCV testing when receiving treatment, *n* (%)	199 (82.3)	144 (85.7)	55 (76.4)
Need HCV medical support when receiving treatment, mean (SD)	7.1 (2.9)	7.2 (2.8)	6.7 (3.2)
Barriers preventing patients from using these services			
Worry that others know about the infection, mean (SD)	3.6 (3.6)	3.8 (3.6)	3.1 (3.6)
Not engaging in risky behavior, will not suffer from HCV, mean (SD)	2.6 (3.3)	2.3 (3.1)	3.4 (3.8)
No physical discomfort, do not need to treat HCV infection, mean (SD)	3.2 (3.5)	2.8 (3.3)	4.2 (3.7)
Worry about withdrawal is unsanitary, so do not test, mean (SD)	2.6 (3.1)	2.19 (2.98)	2.73 (3.52)
Cannot afford treatment, *n* (%)	184 (77.3)	133 (79.2)	51 (72.3)
Cannot find HCV treatment sites, *n* (%)	132 (55.2)	98 (58.3)	34 (47.9)
Doctors cannot comply with the orders, adhere to long-term treatment, *n* (%)	111 (46.4)	80 (47.6)	31 (43.7)
Do not have enough time, *n* (%)	139 (57.9)	96 (57.1)	43 (59.7)
Satisfied with HCV testing service, mean (SD)	7.7 (2.7)	7.5 (2.7)	8.1 (2.7)
Satisfied with HCV education in current treatment site, mean (SD)	7.0 (2.6)	6.9 (2.5)	6.8 (2.9)
Satisfied with medical support, mean (SD)	7.4 (2.7)	7.2 (2.7)	7.7 (2.6)

### MMT staff characteristics and HCV-related services provided in their clinics

MMT staff knowledge of HCV was poor, with an average score of 10.9 (3.1). The most common education services provided in MMT clinics are individual counseling and booklets. In total, 39.7 and 35.6% of staff reported providing education services via group counseling and video programs, respectively. More than 60% of staff reported providing pre- and post-HCV test counseling in their routine work; however, only 31.1% reported providing further testing services, such as PCR or genotype testing. HCV-related medical service was sub-optimal: 45.6% of staff reported providing information on side effect management to patients and 30.5% provided referral services for HCV-positive patients. Other medical treatment services were rare in their routine work. The situation of HCV-related services provided is summarized in Table 4.

**Table 4:** MMT staff characteristics and HCV services provided in their clinics

	Total (*n* = 59)
Males, mean (SD)	41 (69.5)
Age, mean (SD)	46.7 (8.5)
Time of work in MMT clinics (months), mean (SD)	45.3 (20.5)
Education, *n* (%)	
College and higher	14 (24.1)
Less than college	44 (75.9)
Received HCV-related training	10 (16.9)
HCV knowledge scores	10.9 (3.1)
HCV education provided in their sites, *n* (%)	
Provided group counseling	23 (39.6)
Provided individual counseling	36 (62.1)
Provided TV/video program	20 (35.7)
Provided booklet	34 (58.6)
HCV testing provided in their sites, *n* (%)	
Pre-test counseling	35 (60.0)
Post-testing counseling	41 (69.5)
HCV antibody testing	45 (76.3)
Provided genotype/PCR testing	18 (31.1)
HCV medical treatment provided in their sites, *n* (%)	
Have psychical doctors	8 (13.5)
Provided referral service	18 (30.5)
Provided information for liver transplant	5 (8.6)
Provided information for antivirus treatment	9 (15.5)
Help patients deal with side effects	26 (45.6)
Set up group support for HCV-positive patients	8 (13.8)
Case management for HCV-positive patients	10 (17.5)

## Discussion

### Main findings of this study

To the best of our knowledge, this study was the first to investigate HCV knowledge, related services provided, and barriers against HCV-related services for both patients and staff in MMT clinics in China. In the current study, 70% of the participants were HCV positive; thus, the infection rate was significantly higher than in two other studies conducted in MMT clinics 3 years ago (57.0 and 51.3%, respectively) [7, 16]. Consistent with previous studies, many HCV-positive patients were not aware of their serostatus due to limited knowledge and the long duration of clinical progression of HCV infection. There were no epidemiological or longitudinal data; thus, we could not determine the reasons for the increasing HCV infection rate, but the sharp increase of infection rate shows us that long-term follow-up is necessary to monitor changes in patients’ HCV status.

Regarding HCV knowledge, similar to other studies conducted in China and the western countries, both patients and staff had limited HCV knowledge. The patient HCV knowledge score was the same as a previous study in China [16], whereas it was lower than that reported in a US study, which had a mean score of 11.1 (4.1) [21]. The extremely poor HCV knowledge of the MMT staff (mean score of 10.9 (3.1)) is alarming because given individual’s perceptions about drug-related risks are determined in part by their knowledge. The limited knowledge would be a greater barrier for patients accessing HCV-related services and can also decrease staff’s self-confidence to provide related services. Similar to a previous study, needle sharing rates were fairly low compared to other regions in China [7, 8].

Unprotected sexual behavior was confirmed in this study; almost 10% of patients reported having had multiple sexual partners, and more than 10% reported not using condoms with casual or commercial partners. The primary route of HCV transmission was through infected blood; it remains controversial whether hepatitis C can be transmitted through sexual activity. However, condom use was also recommended to prevent HCV transmission, particularly in those with multiple partners who might have a higher risk of erosion to the genitalia. Because drug users are a high-risk population for HCV transmission, protective sexual behavior education should be provided in the prevention program.

Other risk factors that will potentially accelerate the progress to severe liver disease in HCV-positive patients, including alcohol consumption and co-infection of HCV and HBV, have been identified in this study. These findings suggest that individual interventions should be provided to HCV-positive patients in MMT clinics.

### Barriers preventing patients from using HCV-related services

As shown in Table 3, patients expressed the needs to receive HCV-related education, testing, and medical support when they receive treatment, although there were no differences between HCV-positive and HCV-negative patients. However, the patients also described their attitude regarding the barriers that may prevent them from using current services. More than three fourths of the patients reported that the cost of medical treatment prevented them from receiving medical treatment. Other barriers existed, such as difficulty in finding HCV treatment institutes and lacking time to receive treatment. In China, most drug users were not employed and thus did not have social health insurance. Therefore, even if they had the motivation to access medical treatment, economic barriers remained. These barriers prevented patients from accessing therapy, particularly the combination of multiple barriers in the system, which indicates that clinical interventions may not be enough to change the situation; therefore, the government should make more effective measures to improve access.

### HCV-related services in MMT treatment

Overall, the results were not optimistic regarding the HCV knowledge of MMT staff and related services provided in their treatment site. A few more issues were present in this study. It is difficult for staff with limited education who are relatively older to learn new intervention skills. The average time spent working in MMT clinics was 45.3 (20.5) months; however, only 16.9% of staff reported received HCV-related training. Given the limited resources of these clinics, it is difficult for them to provide HCV education and counseling to patients during their routine work. More than half of the staff reported providing pre- and post-test counseling to patients, but most HCV-positive patients were not aware of their HCV status, which indicated that standard HCV testing counseling was not delivered to the patients. From our qualitative research, we found that there was no uniform procedure to inform patients of their HCV test results even though the HCV antibody test was a routine screening test in the MMT clinics. For example, a patient stated that “withdraw blood when I get the treatment, but nobody told me why”, “staff did not tell me the test result, they posted it on the blackboard, I do not understand the meaning of positive and negative”. Limited HCV service for drug users has been addressed in other studies as well. The barriers come from both individuals and the entire system and include the limited resources in MMT clinics, the overload of patient cases, and limited welfare for patients. HCV intervention should be a “combination intervention,” that is, it should involve all possible resources; when all these services are combined in MMT clinics, the combination intervention will benefit the patients [22].

We acknowledge a number of limitations in this study. First, the patients in our study were recruited from Shanghai MMT clinics and thus may not be representative of patients in other areas. Second, we could not exclude the possibility of self-reported bias in the drug users. Finally, there may be other barriers or facilities that prevent or enhance the HCV service that we did not examine in the current study. Future work will be conducted to clarify these issues.

## Conclusions

Our study indicated that HCV infection remains an important problem among MMT patients in China. Barriers for receiving HCV-related services come from both the individual and the entire system. Effective intervention models must urgently be implemented to control HCV infection and associated consequences in China.

## Abbreviations

AIDS: Acquired immune deficiency syndrome
AUDIT: The Alcohol Use Disorder Identification Test
BDI: Beck Depression Inventory
DSM-IV: Diagnostic and Statistical Manual IV
HCV: Hepatitis C virus
HIV: Human immunodeficiency virus
IDU: Injection drug use
MMT: Methadone maintenance treatment
NIDA: National Institute on Drug Abuse
RBS: Risk behaviors survey
SPSS: Statistical Package for the Social Science
WHO: World Health Organization
